# Real-Life Pharmacokinetics of rFVIII-Fc and rFIX-Fc

**DOI:** 10.1055/s-0040-1718416

**Published:** 2020-11-08

**Authors:** Marissa J.M. Traets, Kathelijn Fischer, Nanda Uitslager, Paul R. van der Valk, Idske C.L. Kremer Hovinga, Lize F.D. van Vulpen, Roger E.G. Schutgens

**Affiliations:** 1 Van Creveldkliniek, University Medical Center Utrecht, University Utrecht, Utrecht, The Netherlands

Extended half-life (EHL) products have made their way to clinical practice in the treatment of hemophilia A and B. Phase-3 studies indicated a significantly prolonged terminal half-life (T1/2) of rFVIII-Fc and rFIX-Fc versus standard rFVIII and rFIX; 19.0 versus 12.4 hours (1.5-fold) for FVIII and 82.1 versus 33.8 hours (2.4-fold) for FIX. 1 2 Several studies demonstrated a reduced infusion frequency, lower clotting factor consumption (CFC), and slightly lower annual bleeding rates (ABR). 3 4 5 The objective of this study was to evaluate the impact of switching from standard half-life (SHL) products to rFVIII-Fc (Efmoroctocog alfa, Elocta) and rFIX-Fc (Eftrenonacog alfa, Alprolix) on pharmacokinetics (PK), CFC, prophylactic infusion frequency, and ABR.

This single center observational, retrospective study, conducted at the Van Creveldkliniek in Utrecht, the Netherlands, included patients of all ages who were treated previously with SHL products. The protocol was approved by the local Medical Ethical Committee. Data were collected and analyzed for 24 months before the switch and a mean of 16 months after the switch (range: 4–27 months). Patients were switched between 2016 and 2018. PK assessments (prophylactic dose, minimum three samples) were performed for minimum 3 days (FVIII) or maximum 7 days (FIX) using a kaolin-based one-stage (activated partial thromboplastin time) clotting assay for all assessments. 1 2 Based on comparison with central laboratory data during phase-3 study participation, all measurements of Alprolix were multiplied by 2. Data were entered in the WAPPS-Hemo program, and balanced estimates of terminal half-life, time to reach 0.01 IU/mL (1%) FVIII/IX activity, area under the curve (AUC), clearance, and Cmax were extracted. 6 Prophylactic infusion frequencies were compared during 3 months before the transition and the last 3 months of observation after the transition. Data on CFC were extracted from pharmacy records combined with reported home stocks and patient diaries. The ABR was calculated based on the full follow-up period. Data were analyzed using descriptive statistical methods, including means, medians, 95% confidence interval, and inter–quartile ranges (IQR). Groups were compared using the Wilcoxon sign-rank test and paired-samples *t* -test.

**Fig. 1:**
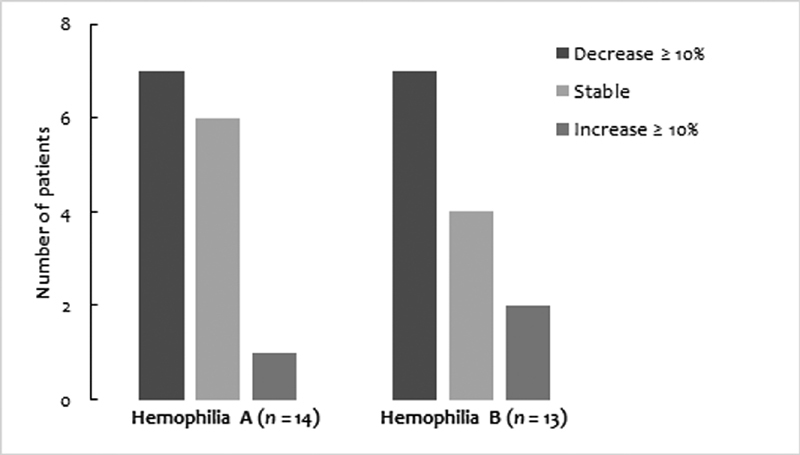
Change in clotting factor consumption.

In total 34 severe hemophilia patients switched to EHL. Four patients were excluded due to immune tolerance induction ( *n* = 1) and missing data ( *n* = 3). Eventually, 30 patients were analyzed, including 50% with hemophilia A. Median age was 35 years (range: 5–79), including 11 children (<18 years). Seven patients (six hemophilia A) had a history of inhibitors. Treatment characteristics and bleeding before and after switching to EHL are shown in Table 1 . T1/2 of rFVIII-Fc was extended 1.4-fold compared with rFVIII ( *p* = 0.004). T1/2 of rFIX-Fc was extended 2.6-fold compared with rFIX ( *p* = 0.005). A subgroup analysis showed shorter T1/2 for both rFVIII-Fc and rFIX-Fc in ex-inhibitor patients, with comparable half-life extension. Extension of T1/2 was age dependent: in children, T1/2 was extended 1.3-fold for rFVIII-Fc and 2.1-fold for rFIX-Fc versus 1.6- and 2.7-fold, respectively, in adults. The median time above 1% FVIII/IX activity after a prophylactic infusion was extended by 1.5 days for hemophilia A ( *p* = 0.002) and by 10.2 days for hemophilia B ( *p* = 0.005). After switching to EHL concentrates, the mean annual CFC dropped 10% in hemophilia A and 29% in hemophilia B patients, *p* = 0.04 (absolute numbers in Table 1 ). A clinically relevant reduction of ≥10% was seen in 7 of 14 hemophilia A and in 7 of 13 hemophilia B patients ( Fig. 1 ). Children showed a more pronounced reduction in CFC than adults, especially children with hemophilia B. Prophylactic infusion frequency was reduced in a minority (4/15: 26.7%) of hemophilia A patients and in almost all (14/15, 93.3%) patients with hemophilia B, who were all able to infuse rFIX-Fc once weekly. ABR decreased in adults with hemophilia A only, from 4.0 to 2.1, ( *p* = 0.05). For patients with hemophilia B, a trend toward ABR reduction was observed in children only, from 3.5 to 2.5, ( *p* = 0.08).

**Table 1:** Treatment characteristics and bleeding before and after switching to FVIII-Fc or FIX-Fc

	Hemophilia A ( *n* = 15)		Hemophilia B ( *n* = 15)	
	Median (IQR)				
Age (y)	29 (6–62)		39 (17–51)	
<18 years	40%		33%	
Blood group O	53%		53%	
	Preswitch	Postswitch	Preswitch	Postswitch
Duration of follow-up (mo)	24	16 (13–21)	24	16 (11–23)
Prophylactic dose/kg/infusion	16 (13–29)	16 (13–29)	26 (15–36)	36 (16–60)
Terminal half-life (h)	10.1 (8.8–12.9)	14.0 (11.5–18.3) a	38.7 (31.3–39.3)	101.4 (78.3–109.8) a
Estimated time to 1% (d)	2.7 (2.5–3.4)	4.2 (3.3–5.3) a	7.2 (6.6–7.8)	17.4 (14.3–21.1) a
AUC (IU∗h/L)	7,863 (6,589–10,678)	13,629 (10,334–17,069) a	9,373 (7,111–10,403)	16,400 (15,257–19,718) a
Clearance (mL/h/kg)	3.67 (2.73–4.25)	2.14 (1.81–3.02) a	4.84 (4.00–5.34)	2.48 (2.22–3.14) a
Cmax (IU/mL)	0.77 (0.52–1.06)	0.75 (0.65–0.97)	0.40 (0.29–0.48)	0.58 (0.54–0.82) a
Number of infusions per week	3.0 (3.0–3.5)	3.0 (2.3–3.5)	2.0 (2.0–3.0)	1.0 (1.0–1.0) a
Annual bleeding rate	2.3 (0.9–7.5)	1.9 (0.6–4.1)	3.5 (0.8–7.8)	2.5 (0.3–3.6)
	Mean (95% confidence interval)			
Annual CFC (IU/kg/year)	3,578 (2,655–4,500)	3,205 (2,414–3,996) a	3,069 (1,768–4,370)	2,182 (1,527–2,836) a

Abbreviations: AUC, area under the curve; CFC, Clotting factor consumption; IQR, interquartile range.

a *p* < 0.05.

This study demonstrates that the switch resulted in an extension of the terminal half-life of rFVIII-Fc (1.4-fold) and rFIX-Fc (2.6-fold) and a significant prolongation of the time to reach a trough level of 1% (1.5 days for hemophilia A and 10.2 days for hemophilia B) with a higher AUC. The infusion frequency of patients with hemophilia A remained stable. The resulting higher and more stable trough levels are expected to provide better protection against bleeds. The A-LONG phase-3 study in hemophilia A included 28 patients with hemophilia A >12 years who underwent PK assessments compared with 15 patients in our study (including 5 <12 years). 1 The T1/2 increase of rFVIII-Fc in our real-life study (1.4-fold) was comparable with the phase-3 study (1.5-fold), as was the time to reach a trough level of 1% (4.2 vs. 4.9 days). For hemophilia B, the B-LONG phase-3 study included 22 patients aged >12 years who underwent PK assessments compared with 15 patients in our study (including 2 <12 years). 2 The T1/2 increase of rFIX-Fc in our real life experience (2.6-fold) was similar to the phase-3 study (2.4-fold). Although the time to reach a trough level of 1% appeared longer in our study (17.4 vs. 11.2 days). This may be explained by differences in modeling and/or the shorter sampling time: 168 versus 240 hours in the phase 3-study. In patients <18 years, T1/2 observed (76.1 hours) was similar to the kids B-LONG study (68.6 hours). 7

Our switching protocol was prespecified and included standard testing, using prophylactic dosing to reflect the real-life experience and patient relevant outcomes. The present study had much longer follow-up than other real-life studies by Keepanasseril et al (6 months pre- and postswitch) 3 and Wang and Young (1 year pre switch, 230 days postswitch) 4 and reported both clinical and PK parameters.

In conclusion, this is the first real-life study reporting on both PK and clinical effects of rFVIII-Fc and rFIX-Fc. We observed significant half-life extension, similar to the phase-3 studies, together with a clear reduction in weekly infusion frequency in hemophilia B and lower annual CFC in both hemophilia A and B.
